# Cell-free synthesis of functional phospholipase A1 from *Serratia* sp.

**DOI:** 10.1186/s13068-016-0563-5

**Published:** 2016-07-29

**Authors:** Hye Jin Lim, Yu Jin Park, Yeon Jae Jang, Ji Eun Choi, Joon Young Oh, Ji Hyun Park, Jae Kwang Song, Dong-Myung Kim

**Affiliations:** 1 Department of Chemical Engineering and Applied Chemistry, Chungnam National University, Daejeon, 305-764 Republic of Korea; 2 Research Center for Bio-based Chemistry, Korea Research Institute of Chemical Technology, Daejeon, 305-600 Republic of Korea

**Keywords:** Industrial enzymes, Biodiesel, Phospholipase A1, Cell-free protein synthesis, Enzymatic degumming

## Abstract

**Background:**

Phospholipase A1 is an enzyme that hydrolyzes phospholipids at the *sn*-1 position. It has potential applications across diverse fields including food, pharmaceutical, and biofuel industries. Although there has been increasing interest in the use of phospholipase A1 for degumming of plant oils during biodiesel production, production of recombinant phospholipase A1 has been hampered by low efficiency of gene expression and its toxicity to the host cell.

**Results:**

While expression of phospholipase A1 in *Escherichia coli* resulted in extremely low productivity associated with inhibition of transformed cell growth, drastically higher production of functional phospholipase A1 was achieved in a cell-free protein synthesis system where enzyme expression is decoupled from cell physiology. Compared with expression in *E. coli*, cell-free synthesis resulted in an over 1000-fold higher titer of functional phospholipase A1. Cell-free produced phospholipase A1 was also used for successfully degumming crude plant oil.

**Conclusions:**

We demonstrate successful production of *Serratia* sp. phospholipase A1 in a cell-free protein synthesis system. Including the phospholipase A1 investigated in this study, many industrial enzymes can interfere with the regular physiology of cells, making cellular production of them problematic. With the experimental results presented herewith, we believe that cell-free protein synthesis will provide a viable option for rapid production of important industrial biocatalysts.

**Electronic supplementary material:**

The online version of this article (doi:10.1186/s13068-016-0563-5) contains supplementary material, which is available to authorized users.

## Background

Increasing demand for sustainable energy has accelerated research and development in biodiesel as well as other sectors of the biofuel industry [1]. Crude oils extracted from plants contain a variety of non-triglyceride components, which need to be removed before the oils can be used as feedstock for biodiesel production.

Degumming is a common practice for the removal of phospholipids before vegetable oils are fed into the biodiesel production process [2]. Phospholipid removal is of particular importance during enzymatic biodiesel production because phospholipids substantially inhibit enzymatic transesterification reactions and decrease the efficiency of biodiesel separation [3, 4]. While hydratable phospholipids can be readily removed by the simple water degumming process, removal of nonhydratable phospholipids typically requires a chemical refining process that usually consists of sequential acid and alkali treatment steps [5]. The chemical degumming process, however, produces large amounts of soapstock, which causes significant loss of oil during its separation. The use of caustic chemicals also has negative impacts on the environment. In this respect, phospholipase A1 (PLA1)-mediated enzymatic degumming to convert nonhydratable phospholipids into hydrophilic lysophospholipids is considered to be a simpler, more efficient and environment-friendly alternative to the chemical processes [6, 7]. Although there are a few commercially available PLA1 sources, relatively little effort has been directed toward the development of bacterial phospholipases for industrial enzymatic degumming. Because plant oils differ substantially in their composition and phospholipid content, it is highly desirable to have a technological platform to produce and characterize phospholipases from diverse sources.

Unlike the conventional cell-based gene expression methods, cell-free protein synthesis uses protein synthesis machinery in open environments and thus can produce proteins by direct addition of template DNA. Therefore, it offers a much faster and more flexible route to protein production [8]. In addition, cell-free protein synthesis does not require the maintenance of cell viability and integrity of cellular membranes. These unique features make cell-free protein synthesis a potentially ideal platform to produce recombinant enzymes that otherwise would interfere with the normal physiology of host cells.

In this study, the technique of cell-free protein synthesis was applied to the expression of functional phospholipase A1 (PLA1) from *Serrartia* sp. While significant amounts of recombinant PLA1 were not obtained from liter-scale cultures of *Escherichia coli* (*E. coli*) due to substantial inhibition of cell growth and protein biosynthesis, cell-free synthesis reactions at microliter scales produced functional PLA1 in amounts sufficient for subsequent analyses. PLA1 synthesized in the cell-free system was confirmed to be highly functional and successful at degumming crude sesame oil. Furthermore, the cell-free synthesis reaction was proven to be readily scalable, showing constant volumetric productivity when scaled-up from 150 µL to 500 mL. As expected, when our phospholipase A1 was utilized to degum crude oil, the cloudy oil was converted to clear. These results demonstrate that cell-free synthesis provides a versatile platform for the production of functional phospholipases, and it can be extended to the production of other industrially important enzymes from analytical to preparative scales.

## Methods

### Chemicals and enzymes

Sesame crude oil was purchased from the market (Daejeon, Korea). Luria-Bertani (LB) medium and ampicillin were purchased from Duchefa Biochemie (Haarlem, The Netherlands). Ni-NTA agarose resin was from Qiagen (Hilden, Germany). ATP, GTP, UTP, CTP, and creatine kinase were purchased from Roche Applied Science (Indianapolis, IN). Creatine phosphate was a kind gift from Bioneer Co. (Daejeon, Korea). L-[U-^14^C] leucine was from Perkin Elmer (Waltham, MA). All other chemical reagents were obtained from Sigma-Aldrich (St Louis, MI) and used without further purification. The S12 extract prepared from the *E. coli* strain BL21Star (DE3) (Invitrogen, Carlsbad, CA) was used as a source of protein synthesis machinery as described previously [9].

### Expression of *Serratia* PLA1 in *E. coli*

The PLA1 gene was PCR-amplified from genomic DNA of *Serratia* sp. MK1 [10] and cloned into the pET21a and the pSTV28 plasmids. The resulting plasmids (pET21a Serr PLA1 and pSTV28 Serr PLA1) was transformed into the *E. coli* strain BL21Star (DE3). After overnight incubation of the seed culture in 5 mL of LB, 4 mL each of the resulting bacterial culture was transferred into 400 mL of LB medium (50 µg/mL of ampicillin or 25 µg/mL of chloramphenicol was used as the antibiotic for each transformant) in two 3 L flasks and grown at 37 °C with vigorous shaking. Expression of PLA1 was induced with 0.6 mM isopropyl β-D-1-thiogalactopyranoside (IPTG) when the OD_600_ of culture broth reached 0.6. Samples were taken throughout the culture period for the assay of expressed enzyme after disruption of the cells by sonication.

### Cell-free synthesis of *Serratia* PLA1

The plasmid pET21a Serr PLA1 used for *E. coli*-based expression of *Serratia* PLA1 was also used as the template for the cell-free protein synthesis reactions. The standard reaction mixture for cell-free synthesis consists of the following components in 150 µL: 57 mM HEPES–KOH (pH 7.5); 1.2 mM ATP; 0.85 mM each of GTP, UTP, and CTP; 80 mM ammonium acetate; 34 µg/mL 1-5-formyl-5,6,7,8-tetrahydrofolic acid (folinic acid); 1.0 mM each of the 20 amino acids; 2 % PEG (8000); 3.2 U/mL of creatine kinase; 67 mM creatine phosphate; 0.01 mM L-[U-^14^C] leucine (11.1 GBq/mmol); 27 % (v/v) of the S12 extract; and 13.0 μg/mL of plasmid. After incubation of the reaction mixture at 37 °C for 3 h, cell-free synthesized protein was quantified by measuring trichloroacetic acid (TCA)-insoluble radioactivity using a Tri-Carb 2810TR liquid scintillation counter (PerkinElmer, Waltham, MA), as described previously [8, 11]. Cell-free synthesized proteins were also analyzed on 12 % SDS-PAGE gels. Enzymatic activity of the PLA1 protein produced during cell-free synthesis reactions was determined using EnzChek^®^ Phospholipase A_1_ Assay Kit (Invitrogen, Carlsbad, CA) following the manufacturer’s protocols.

### Purification of recombinant PLA1

For purification of the PLA1 expressed in *E. coli*, cells were harvested from 1 L of culture broth at 16 h after the addition of IPTG, washed with 500 mL of phosphate buffered saline (PBS) and resuspended in 20 mL of 50 mM sodium phosphate buffer containing 300 mM NaCl and 10 mM imidazole (pH 8.0). The cells were disrupted by sonication on ice. After removing cell debris by centrifugation (10,000 RCF, 15 min), the supernatant was mixed with a 1 mL suspension of Ni-NTA agarose resin. After incubation for 30 min with gentle agitation, the Ni-NTA agarose resin was washed thrice with 100 mL of buffer A (50 mM NaH_2_PO_4_, 300 mM NaCl, 20 mM imidazole, pH 8.0). Subsequent to the final wash, bound proteins were eluted with 5 mL of buffer E (50 mM NaH_2_PO_4_, 300 mM NaCl, 250 mM Imidazole, pH 8.0) and dialyzed against 1 L of 10 mM sodium phosphate buffer (pH 7.4) at twice. For the purification of cell-free synthesized PLA1, 3 mL of a completed cell-free synthesis reaction was centrifuged at 10,000 RCF for 15 min and the supernatant was mixed with 1 mL suspension of Ni-NTA agarose resin. After incubation for 30 min, the beads were washed thrice with 2 mL of buffer A. Subsequent to the final wash, bound protein was eluted with 5 mL of buffer E and twice dialyzed against 1 L of 10 mM sodium phosphate buffer (pH 7.4).

### Degumming of crude plant oils using cell-free synthesized PLA1

Ten mL of crude sesame oil was heated to 60 °C in a water bath for 1 h and cooled down to 40 °C. Varying amounts of purified PLA1 was diluted in 0.5 mL of deionized water and added to the sesame oil. The PLA1 enzyme was dispersed in oil/water emulsion by vortexing (3000 rpm, 1 min) the mixture. The degumming reaction was conducted by placing the tube in an incubating shaker set at 300 rpm and 40 °C. After 12 h of incubation, the degumming reaction was stopped by heating the reaction mixture to 90 °C for 10 min. 0.4 mL of samples were taken and analyzed using a Rxi^®^-5 Sil MS column (Restek, Bellefonte, PA) in a GC7890A gas chromatography analyzer (Agilent Technologies, Santa Clara, CA) connected to a 5975c mass selective detector (MSD). The oven was held at 80 °C for 2 min, ramped to 140 °C at a rate of 10 °C/min, to 240 °C at a rate of 4 °C/min, to 300 °C at a rate of 10 °C/min, and then held at 300 °C for 20 min. Helium was used as the carrier gas at a constant flow rate of 1 mL/min through the column. As an indicator of degumming products, concentration of linoleic acid was measured using dodecanoic acid as an internal standard [12].

## Results and discussion

### *E. coli*-based expression of recombinant *Serratia* PLA1

**Fig. 1:**
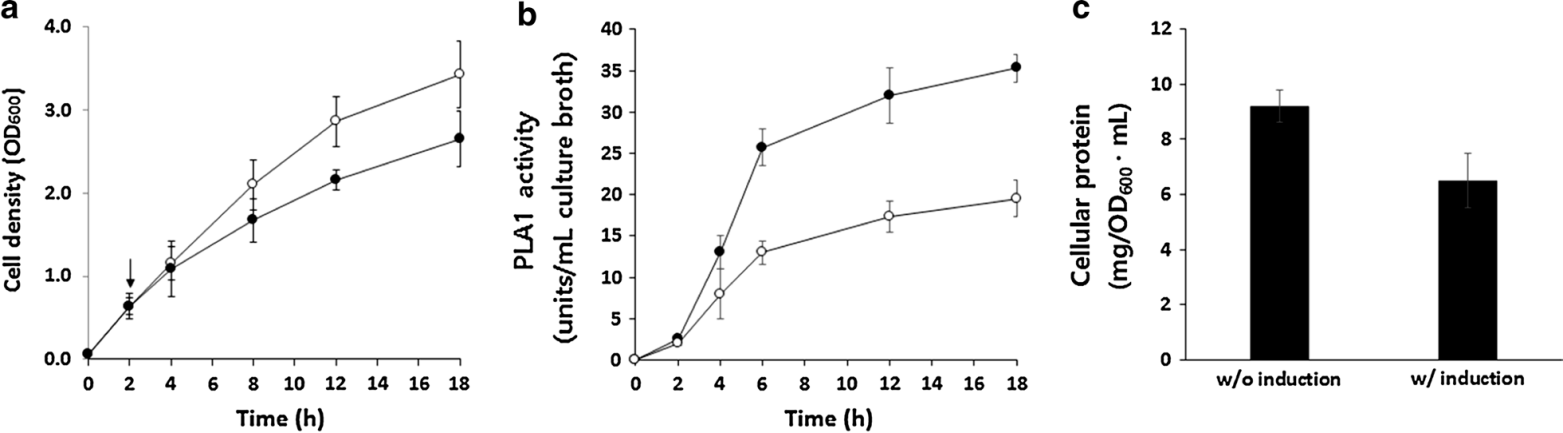
Expression of recombinant PLA1 in *E. coli*. *Escherichia coli* strain BL21 (DE3) was grown in 400 mL of LB media after being transformed with the plasmid pET21a Serr PLA1. Samples of culture broth were taken throughout the culture period to measure optical density at 600 nm (**a**) and PLA1 activity (**b**) as described in the “Methods” section. At 16 h after induction with IPTG, the total amount of cellular protein was measured by the BCA assay (**c**). *Arrows* indicate the time point for IPTG induction. Results from the *E. coli* cultures with or without IPTG induction are shown in *closed* and *open circles*, respectively. *Error bars* represent the standard deviation from three independent experiments

We previously reported very low expression levels of recombinant *Serratia* PLA1 in *E. coli* [13–16]. When the PLA1 gene was expressed under the control of the weak *lac* promoter on a low copy number plasmid (pSTV28), approximately 100 µg of purified PLA1 enzyme was obtained from a 100 mL culture of transformed *E. coli*. It should be noted that the expression level of PLA1 was even lower when the same gene was moved to an expression vector containing a strong promoter. For example, when cloned in the pQE70 plasmid under the T5 promoter, less than 30 µg of purified PLA1 was obtained from a 100 mL culture. Although not discussed in the previous reports, this appears to be due to the toxicity of the enzyme to *E. coli*. Because the T7 promoter used in this study is stronger than the T5 promoter [17–19], we expected that the induction of PLA1 expression would further decrease the yield of PLA1. Indeed, during the cultivation of *E. coli* transformed with the plasmid pET21a Serr PLA1, we found significant growth inhibition after induction with IPTG. A decrease in the growth rate of approximately 25 % was observed in induced *E. coli* as compared to a control culture without IPTG induction (Fig. 1a). Although time-course analysis of PLA1 activity indicated accumulation of functional PLA1 after IPTG induction (Fig. 1b), the expression level was too low to be confirmed by SDS-PAGE and immunoblot analysis (data not shown). We presumed that the onset of PLA1 expression from the strong T7 promoter caused significant damage to the cells and decreased cellular production of proteins. This presumption was confirmed by measuring the amounts of total cellular protein in the *E. coli* with or without IPTG induction. As shown in Fig. 1c, compared to non-induced *E. coli*, the IPTG-induced cells had approximately 25 % less total cellular protein.

Maintenance and alteration of lipid asymmetry are deeply involved in the process of cell division. A number of phospholipid-modifying enzymes are responsible for generating asymmetry in the phospholipid composition of the inner and outer leaflets of cell membranes [20]. One such example is the constitutively expressed outer membrane phospholipase A1 found in *E. coli* and many other gram-negative bacteria [21]. It thus is reasonable to assume that high-level intracellular expression of foreign PLA1 can disturb the balanced asymmetry and deteriorate the integrity of the cell membrane. This assumption is also strengthened by the fact that exogenous PLA1s secreted from pathogenic bacteria, and those found in the venom of bees and snakes, cause destabilization and hemolysis of nearly all kinds of cells [22, 23].

### Cell-free synthesis of PLA1

**Fig. 2:**
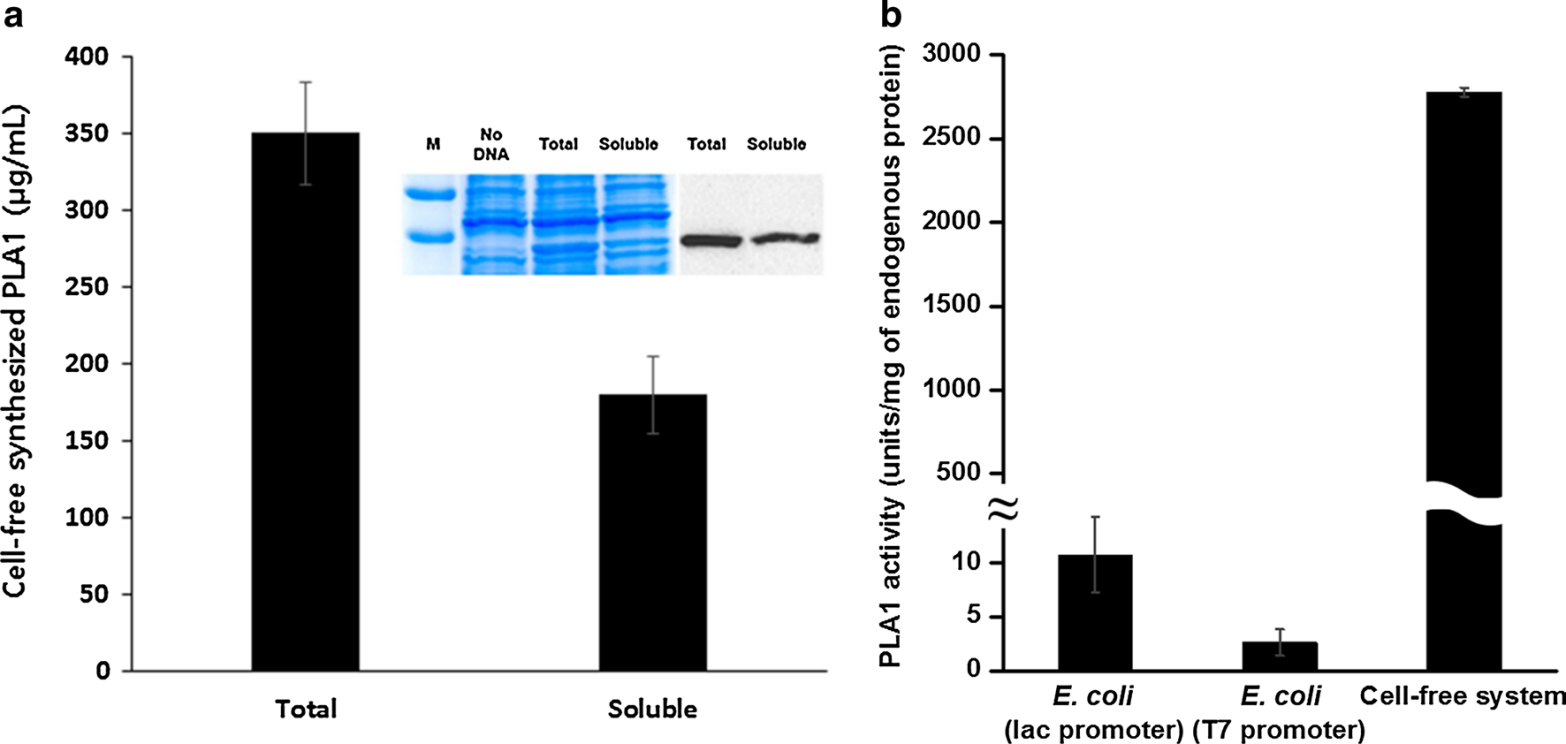
Synthesis of PLA1 from *Serratia* sp. in a cell-free protein synthesis system. The plasmid pET21a Serr PLA1 was incubated in the reaction mixture for cell-free protein synthesis as described in “Methods” section. After incubation for 3 h, total and soluble amounts of cell-free synthesized PLA1 were quantified by measuring the TCA-insoluble radioactivity of the samples (**a**). Embedded image of **a** shows the results of SDS-PAGE and western blot analysis. PLA1 activity produced per mg of endogenous cellular protein in *E. coli* or cell-free protein synthesis system was compared in **b**

Based on the assumption that *E. coli*-based expression of *Serratia* PLA1 is hampered by the membrane toxicity of the expressed enzyme, we decided to investigate the use of cell-free protein synthesis as an alternative expression platform for this enzyme. Because cell-free protein synthesis reactions do not require the presence of an integral membrane, we expected that the enzymatic activity of the synthesized PLA1 would not inhibit continuous expression of this membrane-disrupting enzyme. For a direct comparison, the same plasmid used for the *E. coli*-based expression (pET21a Serr PLA1) was expressed in the cell-free synthesis reaction mixture under the conditions described in “Methods” section. In contrast to the results from the cell-based expression, approximately 0.35 mg/mL of *Serratia* PLA1 was produced after a 3 h incubation of the reaction mixture for cell-free synthesis. In addition, approximately 54 % of the synthesized protein was found in the soluble fraction. Figure 2a shows the results of quantitative measurements of the cell-free synthesized PLA1 along with the SDS-PAGE and immunoblot data. The relative advantage of using the cell-free synthesis system is highlighted when the enzymatic activity of PLA1 was normalized to the amount of endogenous cellular proteins in the S12 extract. As presented in Fig. 2b, PLA1 activity per mg of endogenous protein was more than 1000 fold higher in the cell-free synthesis system than when expressed in *E. coli*. Although PLA1 titer in *E. coli* significantly increased when the expression vector was switched from pET21a (T7 promoter) to pSTV28 (lac promoter), still, normalized enzymatic activity of PLA1 was less than 0.4 % compared to the cell-free synthesis system.

**Fig. 3:**
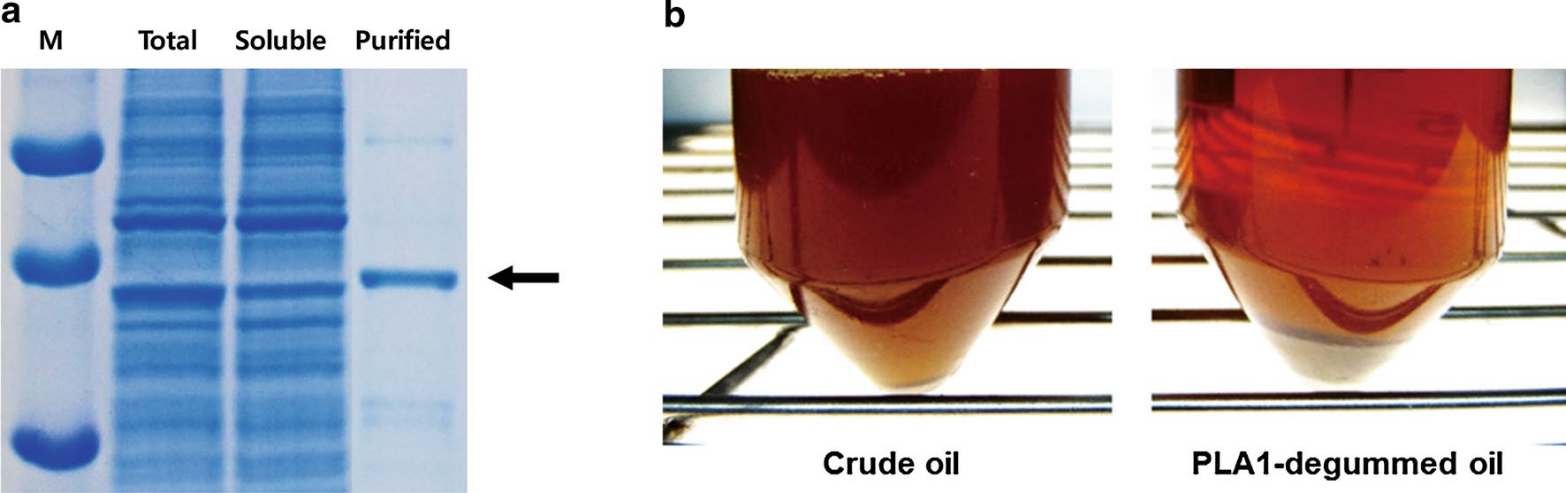
Purification of PLA1 expressed in each system. Cell-free synthesized PLA1 from a 3 mL reaction was purified through a Ni-NTA column (**a**). Turbidity of the crude sesame oil was cleared after incubation with the purified PLA1 (30 µg PLA1 in 10 mL oil) for 12 h at 40 °C, indicating that the cell-free synthesized enzyme catalyzed degumming of crude oil (**b**)

While we failed to obtain meaningful amounts of purified PLA1 from a 1 L culture of *E. coli*, a clear band of PLA1 was confirmed on the Coomassie blue-stained gel after purification from a 3 mL cell-free reaction (Fig. 3a). It was estimated that approximately 300 µg of purified PLA1 was recovered from the 3 mL cell-free synthesis reaction (recovery yield ≈40 %). The PLA1 enzyme purified from the 3 mL of cell-free synthesis reaction was used for degumming of crude sesame oil to test its usability in biodiesel production. As shown in Fig. 3b, incubation with the cell-free synthesized PLA1 cleared the cloudy crude oil, indicating that the recombinant PLA1 successfully catalyzed the degumming reaction. Gas chromatography analysis results also showed an increase in the concentration of linoleic acid in the oil depending on the amount of the used enzyme (Table 1 and Additional file 1: Figure S1).

**Table 1:** Gas chromatography analysis of crude sesame oil incubated with cell-free synthesized PLA1

Amounts of PLA1 (µg)	Concentration of linoleic acid in oil (mM)
0	8.7
10	16.4
20	21.0
30	28.6

10 mL of crude sesame oil was incubated with varying amounts of cell-free synthesized PLA1 and analyzed as described in “Methods” section

**Fig. 4:**
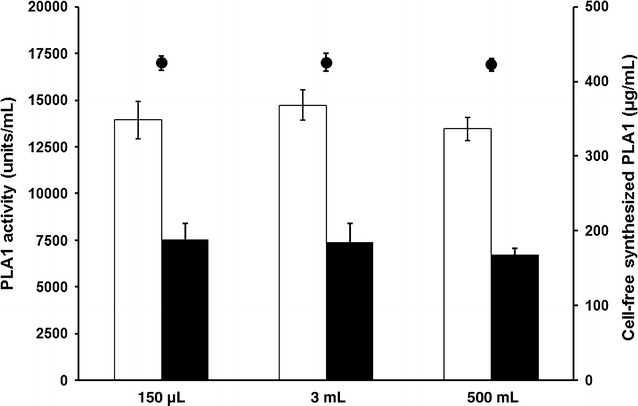
Cell-free synthesis of PLA1 in varying reaction volumes. The reaction scale of cell-free PLA1 synthesis was increased sequentially from 150 µL to 3 mL to 500 mL. The total and soluble productivity and titer of functional PLA1 were not significantly affected by the reaction volume. The *bars* in the graph represent total (*blank*) and soluble (*filled*) amounts of cell-free synthesized protein. *Circles* represent PLA1 activity measured in the reaction mixture. *Error bars* represent the standard deviation from three independent experiments

We next examined whether the reaction volume of cell-free synthesis can be changed without affecting the productivity of functional enzymes. While optimal conditions of heterogeneous cell culture often vary depending on size, the homogenous nature of cell-free protein synthesis reactions results in a more chemistry-like scale-up [24, 25]. When the reaction volume of the cell-free PLA1 synthesis was sequentially increased from 150 µL to 500 mL, as shown in Fig. 4, the volumetric production of functional PLA1 was maintained consistently across the different reaction volumes examined (150 µL, 3 and 500 mL).

## Conclusions

PLA1 has broad applications in diverse industrial sectors [6, 26]. In this study, we demonstrated successful production of *Serratia* sp. PLA1 in a cell-free protein synthesis system. Compared to *E. coli*-based expression, where the production of this enzyme was hampered by damage to cell growth and extremely low levels of expression, efficient production of functional enzyme was achieved using cell-free protein synthesis. Furthermore, degumming of crude oil was successfully performed by the PLA1 synthesized from our cell-free protein synthesis system. Including the PLA1 investigated in this study, many industrial enzymes can interfere with the regular physiology of cells, making their production problematic. With the experimental results presented herewith, we believe that cell-free protein synthesis can be considered as a versatile option for rapid production of important industrial biocatalysts. For now, the presented approach would be most ideal for high-speed screening and characterization of enzymes because the high cost of the reagents can limit its scale-up for industrial production. With the present composition of the reaction mixture for cell-free synthesis, 1 mL of reaction mixture costs approximately 3.5 USD. As demonstrated in Additional file 2: Figure S2, energy source, extract preparation and nucleotide triphosphate account for more than 80 % of total reagent cost. Fortunately, it has been proven that the costs for these components can be markedly reduced by the use of cheap energy sources (glucose, for example), preparation of cell extract after high-cell density culture of *E. coli* cells and use of NMP instead of NTP, respectively [27]. We thus expect that both the productivity and economics of cell-free synthesis of enzymes can be substantially improved for industrial applications in the near future.

## Additional files

10.1186/s13068-016-0563-5 Gas chromatography analysis of sesame oil incubated with cell-free synthesized PLA1.
10.1186/s13068-016-0563-5 Analysis of reagents for cell-free protein synthesis.

## Abbreviations

PLA1: phospholipase A1
LB: Luria-Bertani
IPTG: β-D-1-thiogalactopyranoside
Folinic acid: 1-5-formyl-5,6,7,8-tetrahydrofolic acid
TCA: trichloroacetic acid
PBS: phosphate buffered saline
Ni-NTA: nickel-nitrilotriacetic acid
RCF: relative centrifugal force
RPM: revolutions per minute
SDS-PAGE: sodium dodecyl sulfate-polyacrylamide gel electrophoresis
NMP: nucleotide monophosphate
NTP: nucleotide triphosphate
