# Supplementary material for: Cell-free synthesis of functional phospholipase A1 from Serratia sp
Source: Biotechnol Biofuels. 2016 Jul 29;9:159. doi: 10.1186/s13068-016-0563-5 (PMC4966862; doi:10.1186/s13068-016-0563-5)
Supplement: Supplementary file 1 — 10.1186/s13068-016-0563-5 Gas chromatography analysis of sesame oil incubated with cell-free synthesized PLA1. [file 13068_2016_563_MOESM1_ESM.pdf]

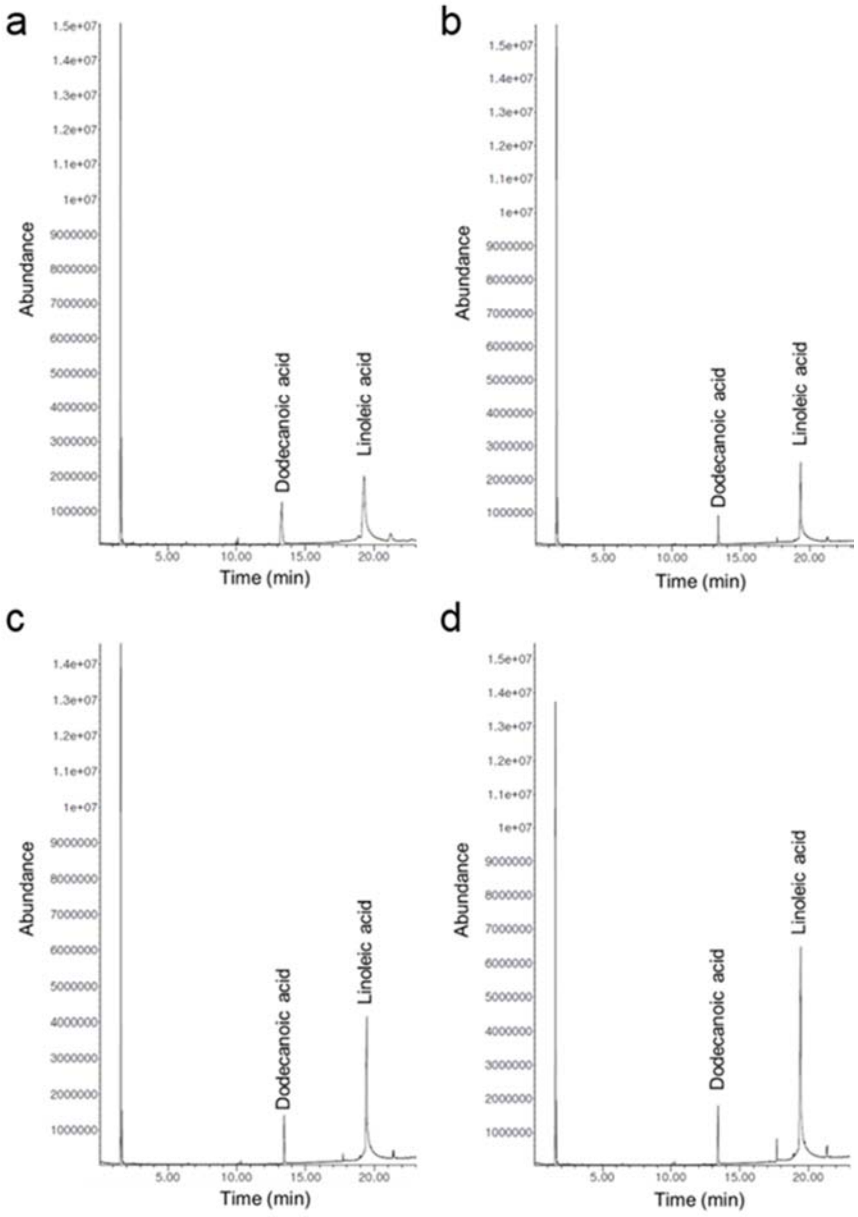


**Figure S1. Gas chromatography analysis of sesame oil incubated with cell-free synthesized PLA1.** 0 (a), 10 (b), 20 (c) and 30 (d) μg of cell-free synthesized PLA1 were added to 10 mL of crude sesame oil. After 12 h incubation, samples were taken and analyzed by gas chromatography as described in Methods.
