# Supplementary figures and images for: Cell-free synthesis of functional phospholipase A1 from Serratia sp
Source: Biotechnol Biofuels. 2016 Jul 29;9:159. doi: 10.1186/s13068-016-0563-5 (PMC4966862; doi:10.1186/s13068-016-0563-5)

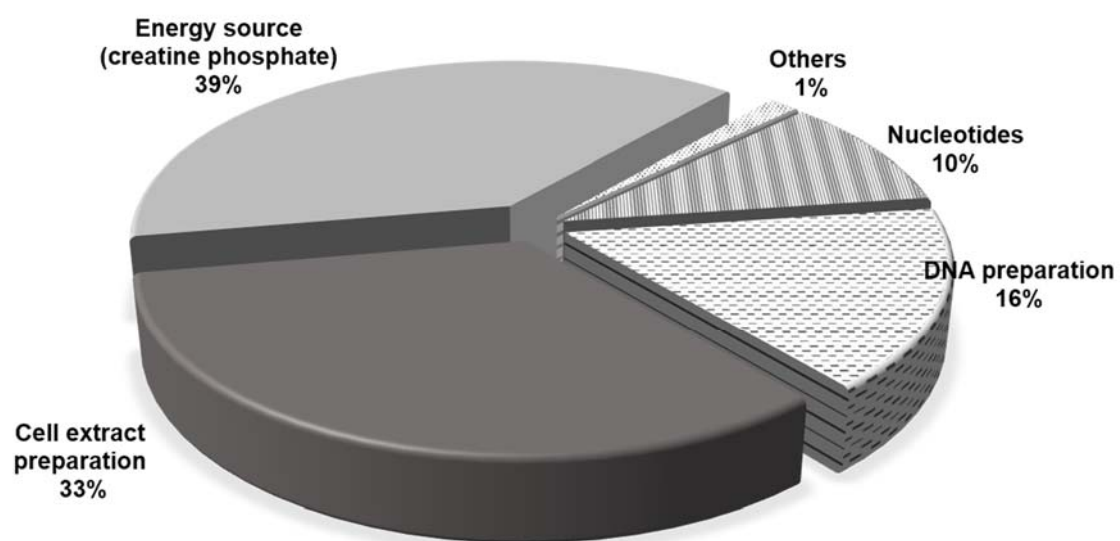

**Figure S2. Analysis of reagent cost for cell-free protein synthesis.**

Supplement: Supplementary file 2 — 10.1186/s13068-016-0563-5 Analysis of reagents for cell-free protein synthesis. [file 13068_2016_563_MOESM2_ESM.pdf]
